# Interleukin-1β Processing Is Dependent on a Calcium-mediated Interaction with Calmodulin*

**DOI:** 10.1074/jbc.M115.680694

**Published:** 2015-11-11

**Authors:** Joseph S. Ainscough, G. Frank Gerberick, Ian Kimber, Rebecca J. Dearman

**Affiliations:** From the ‡ Faculty of Life Sciences, University of Manchester, Manchester M13 9PT, United Kingdom and; the § Procter & Gamble Co., Cincinnati, Ohio 45253

**Keywords:** calcium, calmodulin (CaM), caspase 1 (CASP1), inflammasome, IL-1, IL-1β

## Abstract

The secretion of IL-1β is a central event in the initiation of inflammation. Unlike most other cytokines, the secretion of IL-1β requires two signals: one signal to induce the intracellular up-regulation of pro-IL-1β and a second signal to drive secretion of the bioactive molecule. The release of pro-IL-1β is a complex process involving proteolytic cleavage by caspase-1. However, the exact mechanism of secretion is poorly understood. Here we sought to identify novel proteins involved in IL-1β secretion and intracellular processing to gain further insights into the mechanism of IL-1 release. A human proteome microarray containing 19,951 unique proteins was used to identify proteins that bind human recombinant pro-IL-1β. Probes with a signal-to-noise ratio of >3 were defined as biologically relevant. In these analyses, calmodulin was identified as a particularly strong hit, with a signal-to-noise ratio of ∼11. Using an ELISA-based protein-binding assay, the interaction of recombinant calmodulin with pro-IL-1β, but not mature IL-1β, was confirmed and shown to be calcium-dependent. Finally, using small molecule inhibitors, it was demonstrated that both calcium and calmodulin were required for nigericin-induced IL-1β secretion in THP-1 cells and primary human monocytes. Together, these data suggest that, following calcium influx into the cell, pro-IL-1β interacts with calmodulin and that this interaction is important for IL-1β processing and release.

## Introduction

IL-1β is a potent pro-inflammatory cytokine of the IL-1 cytokine family, functioning as a key mediator in the response to infection and injury (1, 2). The mechanisms involved in IL-1β secretion are of some significance. However, despite considerable interest, the processes involved in IL-1β processing and release are complex and remain poorly understood.

Unlike most other cytokines, IL-1β does not have a signal peptide and, therefore, is not secreted via the classical secretory pathway (3). Instead, secretion is a multistep process, requiring both the up-regulation of the precursor pro-IL-1β and proteolytic cleavage to yield the bioactive molecule. Up-regulation of pro-IL-1β is a well defined process and is typically induced by the detection of pattern-associated molecular patterns by pattern recognition receptors (4, 5). The detection of pattern-associated molecular patterns by pattern recognition receptors drives the induction of complex signaling pathways, culminating in the translocation of NF-κB into the nucleus (6). In turn, NF-κB initiates the transcription of a number of pro-inflammatory proteins, including pro-IL-1β (7).

At this stage, secretion is dependent on the proteolytic cleavage of the 31-kD precursor molecule into its 17-kD bioactive form. Without this step, pro-IL-1β is polyubiquitinated and degraded by the proteasome (8). Pro-IL-1β cleavage is dependent on the activation of caspase-1, which is driven by the assembly of the inflammasome (9, 10). Typically, the inflammasome complex is comprised of an inflammasome sensor molecule, caspase-1, and an adaptor protein called apoptosis speck-like protein containing a CARD (ASC)^2^ (11, 12). There are many known inflammasome sensor molecules, most commonly of the NOD-like receptor (NLR) family, and these serve to detect the presence of an array of pattern-associated molecular patterns and damage-associated molecular patterns (13). The most comprehensively studied NLR is NLRP3 (14). The study of NLRP3 has elucidated a diverse range of stimuli capable of inducing the assembly of the inflammasome, including ATP (15); the crystalline compounds silica (16), asbestos (17), and uric acid (18); the bacterial product listeriolysin O (19); and the potassium ionophore nigericin (20). Given this diversity of stimuli, it is unlikely that a single unifying mechanism for NLRP3 activation exists. Instead, it has been proposed that NLRP3 acts as a sensor of cellular changes induced by certain danger signals (21).

The importance of fluctuating intracellular ion concentrations for the assembly of the NLRP3 inflammasome is well established. Potassium efflux has been observed in response to many NLRP3 stimulants, including ATP, bacterial pore-forming toxins, and nigericin (22). Crucially, Pétrilli *et al.* (23) have demonstrated that NLRP3 inflammasome assembly, caspase-1 activation, and IL-1β maturation were inhibited when potassium efflux was inhibited. It is not clear, however, whether potassium efflux alone is sufficient for inflammasome assembly and IL-1β processing. In addition to potassium, calcium is also implicated in NLRP3-dependent IL-1β secretion. Specifically, ATP and nigericin have both been shown to induce the release of intracellular calcium stores, leading to an increase in cytosolic calcium concentration (24). Importantly, the same study has also demonstrated that the chelation of intracellular calcium inhibits the processing and release of pro-IL-1β in murine macrophages, suggesting that an increase in cytosolic calcium concentration is required for this process. However, despite continuing efforts, the exact role of calcium in IL-1β secretion remains unknown.

Calmodulin is a calcium binding protein that is found in all eukaryotic cells (25). Upon increasing intracellular calcium concentrations, each calmodulin can bind up to four calcium ions via its EF-hand domain (26). These interactions result in a conformational change in the calmodulin, allowing it to bind to its target protein(s). Using a human proteome microarray comprising 19,951 unique proteins to identify those that bind human recombinant pro-IL-1β, we show here, for the first time, that pro-IL-1β binds calmodulin. We also confirmed that this interaction is specific for pro-IL-1β but not mature IL-1β and that it is dependent on the presence of calcium ions. Finally, we show that calcium and calmodulin are required for IL-1β secretion by both the human THP-1 monocytic cell line and primary human monocytes. Taken together, these data provide strong evidence that the direct interaction between calmodulin and pro-IL-1β is pivotal in driving IL-1β processing.

## Experimental Procedures

### 

#### 

##### Antibodies and Reagents

LPS from *Escherichia coli* serotype 055:B5 (Toll like receptors 2/4) and nigericin were purchased from Sigma. The recombinant proteins used were human pro-IL-1β, human calmodulin (both from Sino Biological, Philadelphia, PA), and human IL-1β (R&D Systems, Minneapolis, MN). The calcium chelator BAPTA-AM was purchased from Life Technologies, and the calmodulin inhibitors E6 berbamine and W7 were purchased from Enzo Life Sciences (Exeter, UK) and Santa Cruz Biotechnology, respectively. For Western blot analysis, the primary antibodies used were a goat anti-human IL-1β antibody (R&D Systems) or a rabbit anti-human caspase-1 (p10) antibody (Santa Cruz Biotechnology). The secondary antibodies used were a sheep anti-mouse IgG antibody (AbD Serotech, Kidlington, UK) or a goat anti-rabbit antibody (Dako, Copenhagen, Denmark). For immunofluorescence analysis, the primary antibodies used were a rabbit anti-ASC antibody (Santa Cruz Biotechnology), a rabbit anti-calmodulin antibody (Abcam, Cambridge, UK), or a goat anti-human IL-1β antibody (R&D Systems). The secondary antibodies used were an Alexa Fluor 488-conjugated goat anti-rabbit IgG antibody or an Alexa Fluor 594-conjugated rabbit anti-goat IgG antibody (both from Life Technologies).

##### Identification of Pro-IL-1β-interacting Proteins Using HuProt Human Proteome Microarrays

Two HuProt human protein microarray slides (v.2.0) containing 19,951 probe sets spotted in duplicate were purchased from CDI Laboratories (Mayaguez, PR). Microarray slides were preincubated in block buffer (2% BSA and 0.1% Tween in PBS) for 2 h at room temperature. Slides were then aspirated and incubated with recombinant human pro-IL-1β (10 μg/ml) diluted in reagent diluent (PBS containing Ca^2+^ with 0.1% Tween) or reagent diluent alone (negative control) for 1 h at room temperature. Both slides were aspirated and washed three times with reagent diluent. The microarray slides were then incubated with mouse anti-human IL-1β antibody (R&D Systems) diluted in reagent diluent. As before, slides were aspirated and washed three times. Finally, slides were incubated with an Alexa Fluor 633-conjugated goat anti-mouse secondary antibody (2 μg/ml) in reagent diluent for 1 h at room temperature, followed by a final three washes. The microarrays were spun dry at 1500 rpm for 3 min and scanned using a GenePix 4000B microarray scanner (Molecular Devices, Sunnyvale, CA).

##### Proteome Microarray Analysis

The images acquired by the microarray scanner were analyzed using Genepix Pro 6.0 microarray analysis software (Molecular Devices). Probe signals were acquired from the slides using GenePix Pro 6.0 software (Molecular Devices). The Genepix Pro 6.0 software also calculated noise using an algorithm to determine the background signal of each block. The proteins were considered to be a hit when the average signal-to-noise ratio (SNR) of each duplicate was above 3.

##### Protein Binding Assay

96-well immunoplates (Nunc, Thermo Fisher Scientific, Waltham, MA) were coated with PBS alone or with various concentrations of either BSA or recombinant calmodulin diluted in PBS and incubated overnight at 4 °C. Plates were washed three times with wash buffer (0.05% Tween 20/PBS). Wells were blocked with 1% BSA/PBS for 1 h at room temperature. Plates were washed a further three times, and 3 nm recombinant human pro-IL-1β or recombinant human IL-1β in reagent diluent (PBS with 0.1% tween) was added to the wells. The plates were then incubated for 2 h at room temperature. After another three washes, biotinylated goat anti-human IL-1β (R&D Systems, 200 ng/ml in reagent diluent) was added to the wells, and the plates were incubated for 2 h at room temperature. Again, the plates were washed three times, and 100 μl of streptavidin-HRP (1:40, R&D Systems) diluted in reagent diluent was added to each well. The plates were then incubated in the dark for 20 min at room temperature. Following a final washing step, tetramethylbenzidine-stabilized chromogen (Life Technologies) was added to the wells, and the plates were incubated in the dark for 10 min. The reaction was stopped with 0.5 m sulfuric acid. Plates were read at 450 nm using a BioTek ELX800 plate reader and Gen5 data analysis software (both from BioTeK, Winooski, VT).

To examine whether calcium is a requirement in the interaction between calmodulin and pro-IL-1β, the protein-binding assay was repeated using PBS without calcium in the reagent diluent. To further investigate the role of calcium, 10 mm EDTA or 10 mm EGTA was added for either the final 10 min or the full 2 h of the pro-IL-1β incubation. In experiments designed to investigate the relative affinity of the detection antibody for pro- and mature IL-1β, the plates were coated with the primary antibody from the human IL-1β duoset (R&D Systems). These plates were then processed as described above.

##### Maintenance and Treatment of the THP-1 Cell Line

THP-1 cells were cultured in FCS-supplemented culture medium (RPMI 1640 medium, Life Technologies) containing 400 μg/ml penicillin/streptomycin, 292 μg/ml l-glutamine, 0.05 mm 2-mercaptoethanol, and 10% FCS (Life Technologies). 10^6^ THP-1 cells or 10^7^ THP-1 cells (both 1 × 10^6^ cells/ml) were cultured in 24- or 6-well tissue culture plates, respectively. Cells were primed with LPS (1 μg/ml) for 4 h to induce up-regulation of pro-IL-1β. To induce pro-IL-1β processing and secretion, cells were treated with 10 μm nigericin for another hour. E6 berbamine was added for the final 30 min of THP-1 cell priming. BAPTA-AM and W7 inhibitors were both added 15 min prior to the addition of nigericin. After incubation, supernatants were harvested and frozen at −80 °C. Cell lysates were harvested in 200 μl of lysis buffer (20 mm Tris HCl, 137 mm NaCl, 20 mm EDTA, 10% glycerol, 0.5% Igepal, 1 mm PMSF, and protease inhibitor mixture (1:100)) and frozen at −80 °C.

##### Isolation and Treatment of Human Primary Peripheral Blood Monocytes

Buffy coats from the peripheral venous blood of healthy donors were acquired from the National Health Service Blood and Transplant Service (Manchester Donor Centre, Plymouth Grove, Manchester, UK). The collection and use of this tissue was approved by a blanket material transfer agreement. Peripheral blood mononuclear cells were isolated from whole blood by density centrifugation using Histopaque (Sigma). Monocytes were enriched from peripheral blood mononuclear cells using the negative magnetic selection monocyte isolation kit II (Miltenyi Biotec, Bergisch Gladbach, Germany). Monocyte purity was determined by staining with an allophycocyanin-labeled anti-human CD14 antibody (BD Biosciences) and analysis by flow cytometry (FACSCalibur flow cytometer, BD Biosciences). Monocytes (1 ml of 1 × 10^6^ cells/ml) were cultured in 24-well tissue culture plates in FCS-supplemented culture medium (Iscove's modified Dulbecco's medium, Life Technologies) containing 400 μg/ml penicillin/streptomycin and 10% FCS (Life Technologies). Cells were primed with LPS (1 ng/ml) for 4 h to induce up-regulation of pro-IL-1β. To induce processing and secretion of pro-IL-1β, cells were treated with 10 μm nigericin for another hour. E6 berbamine was added for the final 30 min of cell priming. After incubation, supernatants were harvested and frozen at −80 °C. Cell lysates were harvested in 200 μl of lysis buffer and frozen at −80 °C.

##### ELISA

Supernatants and lysates were analyzed for the presence of IL-1β or IL-8 protein using specific ELISA Duosets from R&D Systems. ELISAs were performed following the instructions of the manufacturer.

##### Western Blot Analysis

In preparation for Western blot analysis, supernatants and lysates were diluted in sample buffer (Bio-Rad) containing 1% 2-mercaptoethanol and heated for 5 min at 80 °C. Samples were resolved on a 10% acrylamide gel, and proteins were transferred to a nitrocellulose membrane. Specific proteins were detected using goat anti-human IL-1β (1 μg/ml) or rabbit anti-human caspase-1 (p10) antibody (0.5 μg/ml). Finally, blots were incubated with a HRP-labeled anti-goat IgG antibody or a HRP-labeled anti-rabbit IgG antibody (both 0.25 μg/ml), and proteins were visualized using enhanced chemiluminescence reagents (Thermo Scientific, Waltham, MA).

##### Immunofluorescence Staining of THP-1 Cells

After treatment as described above, THP-1cells were fixed with 4% formaldehyde for 20 min at room temperature. Cells were then washed with PBS and reconstituted at 5 × 10^5^ cells/ml in PBS. Cells were fixed onto glass slides by transferring 200 μl of the cell suspension into cytospin cartridges and spinning in the cytospin centrifuge at 700 × *g* for 5 min. The glass slides were incubated in 0.2 m glycine (Sigma) for 20 min and then washed with PBS. THP-1 cells were permeabilized by incubating the slides in 0.5% Triton X-100/PBS solution (Sigma) for 8 min at room temperature and blocked in block buffer (3% BSA in PBS) overnight at 4 °C. The slides were then incubated with anti-ASC antibody (5 μg/ml), an anti-calmodulin antibody (1:100), or an anti-IL-1β antibody (5 μg/ml) diluted in block buffer. After washing with PBS, the slides were incubated with Alexa Fluor 488-conjugated goat anti-rabbit IgG antibody or Alexa Fluor 594-conjugated rabbit anti-goat IgG antibody (both 10 μg/ml). Finally, slides were mounted using Vectashield (Vector Laboratories Ltd., Peterborough, UK), sealed with nail varnish, and imaged using fluorescence microscopy. In these experiments, the operator was blinded to the identity of the samples.

##### Statistical Analyses

Statistical analyses were performed using the software GraphPad Prism 6. Data were analyzed by one-way ANOVA to determine overall differences and a Tukey post hoc test was performed to determine statistically significant differences between treatment groups (*, *p* < 0.05; **, *p* < 0.01).

## Results

### 

#### 

##### Identification of Proteins Interacting with pro-IL-1β Using Human Proteome Microarrays

**FIGURE 1.**
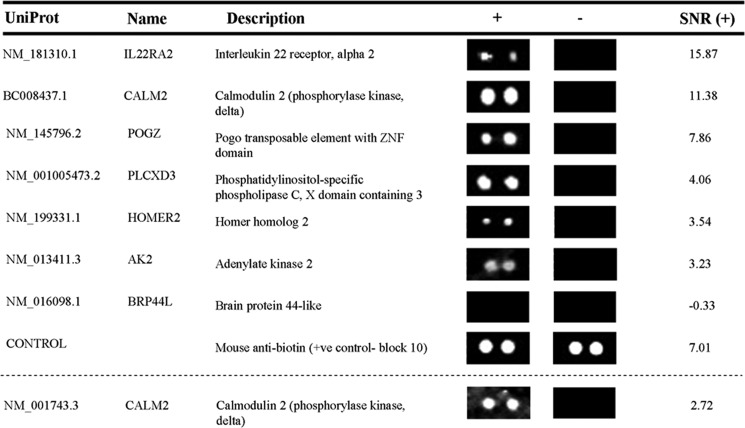
**Identification of pro-IL-1β interacting proteins on a human proteome microarray.** A human proteome microarray was incubated with human recombinant pro-IL-1β (+) or with reagent diluent alone as a negative control (−) and probed with a mouse anti-human IL-1β antibody. An SNR value was generated for each probe set, with an SNR of >3 considered to be a hit. A comprehensive list of all identified pro-IL-1β interacting proteins (hits) is shown, including one representative negative result (for brain protein 44-like) and a positive control (anti-biotin mouse monoclonal IgG). The identity of each protein, the corresponding images for each duplicate set of proteins on the array, and the mean SNR for the duplicate protein spots are shown.

The HuProt microarray was used to assess the interaction between human recombinant pro-IL-1β and 19,951 individual probe sets. The probe sets were full-length recombinant proteins expressed as glutathione-S-transferase-His_6_ fusions in the yeast *Saccharomyces cerevisiae*. In parallel, a microarray was processed without the addition of pro-IL-1β and used as the negative control. Of the 19,951 probe sets, only six qualified as positive hits (displayed an average SNR of >3) and were therefore identified as potential pro-IL-1β-interacting proteins. All hits identified are illustrated in Fig. 1, along with a representative negative control protein (the vast majority of probes were negative) and a representative positive control (each of the 48 blocks on the array contained a positive control protein, an anti-biotin mouse monoclonal antibody that should bind the goat anti-mouse secondary antibody, to confirm the quality of both the IL-1β and control microarrays). Of particular interest in the list of hits were the following: IL-22 receptor α2 (IL-22Rα2), a secreted protein involved in the inhibition of IL-22; phosphatidylinositol-specific phospholipase C (X domain-containing 3) (PLCXD3),an enzyme involved in cell signaling pathways that lead to the release of intracellular calcium; pogo-transposable element containing a zinc finger domain (POGZ), an intracellular protein that has been shown to interact with certain transcription factors; and calmodulin, a ubiquitously expressed, calcium-sensitive messenger protein.

Given the pre-existing links between calcium signaling and IL-1β secretion, and given that calmodulin was such a strong hit on the microarray (SNR, 11.379), the interaction between calmodulin and pro-IL-1β was examined in further detail. Interestingly, the microarray also included an additional probe set representing the same CALM2 gene encoded by a slightly different mRNA sequence (NM_001743.3). Although this probe set scored an average SNR of 2.721 and, therefore, is just below the arbitrary threshold, this SNR is still high and supports data suggesting that calmodulin interacts with pro-IL-1β. Curiously, there are three different genes (designated CALM1, CALM2, and CALM3) that encode for the same protein (27). Because the protein encoded by CALM3 was also present as a probe set on the microarray (BC006182.1), it was of concern that this probe set was negative on the array (SNR, −1.104). However, a closer analysis of the mRNA sequence used for the CALM3 probe set revealed that the protein encoded by this sequence does not share complete protein sequence homology with native calmodulin. Because the CALM2 (BC008437.1) sequence does encode a protein that shares 100% protein sequence homology with native calmodulin, it is likely that the subtle differences in the proteins encoded by CALM2 (BC008437.1) and CALM3 (BC006182.1) cause the observed differences in their capacity to interact with pro-IL-1β. Together, these data provide strong evidence that calmodulin interacts with pro-IL-1β.

##### The Interaction between IL-1β and Calmodulin Is Robust, Specific for the Pro-protein Only, and Dependent on Calcium

**FIGURE 2.**
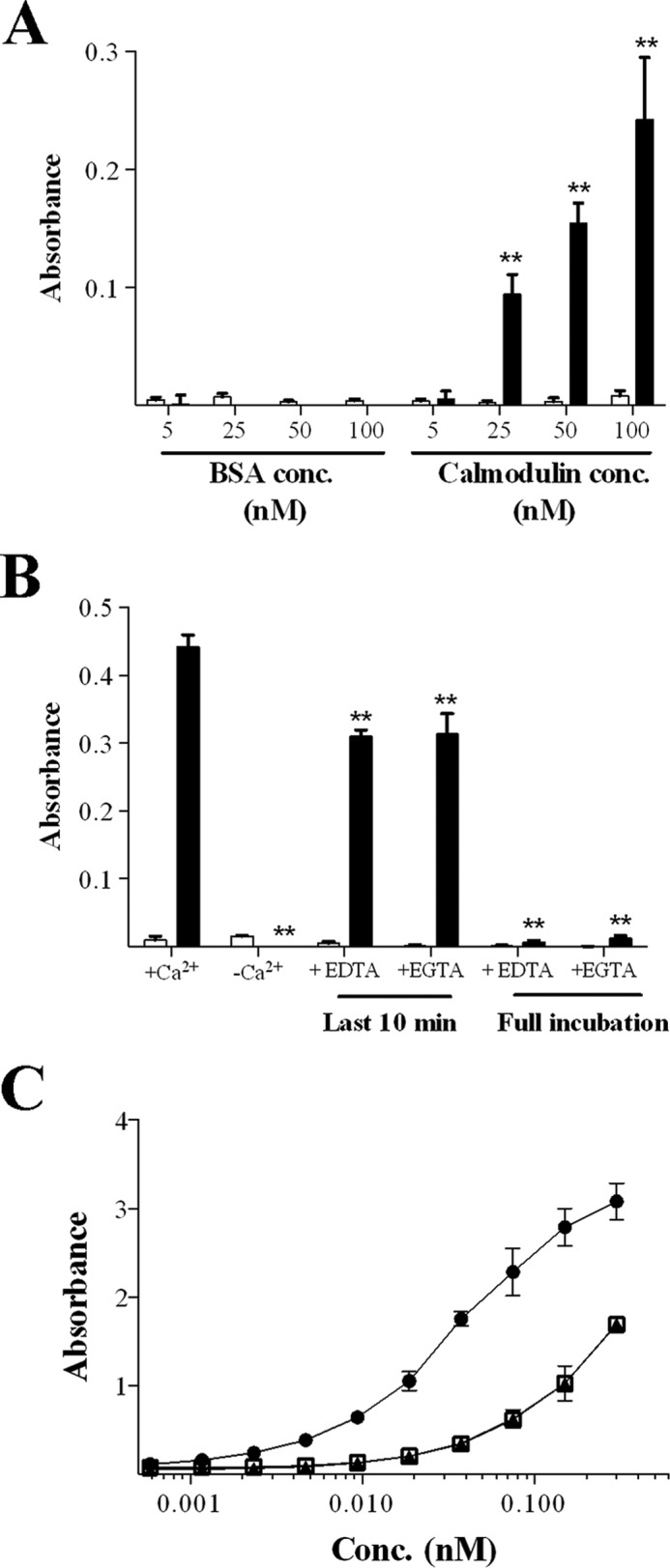
**Calmodulin interacts with pro-IL-1β and not mature IL-1β, and this interaction is dependent on calcium.** Recombinant pro-IL-1β (3 nm, *black columns*) or recombinant mature IL-1β (3 nm, *white columns*) was added to wells of 96-well immunoplates coated with either recombinant calmodulin or BSA control (both at concentrations of 5, 25, 50, or 100 nm). After washing, the amount of IL-1β bound to the wells was detected using a HRP-labeled anti-IL-1β antibody and a chromogenic substrate, with absorbance used as a measure of interaction between the IL-1β and the coating proteins. *conc*, concentration. *B*, the protein-binding assay was repeated using 50 nm of calmodulin (*black columns*) or 50 nm of BSA (*white columns*) and a reagent diluent with and without Ca^2+^ for the duration of the incubation or with and without 10 mm EDTA or EGTA for either the full 2-h incubation or the final 10 min of incubation. *C*, to determine how these treatments affected IL-1β detection by the HRP-labeled anti-IL-1β secondary antibody and to determine the relative affinity of this detection antibody for pro- and mature IL-1β, 96-well immunoplates were coated with an anti-IL-1β antibody and incubated with either recombinant mature IL-1β (●), recombinant pro-IL-1β in standard diluents (□), or recombinant pro-IL-1β diluted in reagent diluent without Ca^2+^(▴) diluted using a 9-fold serial dilution (no differences observed here). The data shown are mean ± S.E. of three independent analyses. The statistical significance of differences between BSA- and calmodulin-treated samples was determined by one-way ANOVA. **, *p* < 0.001 (*A*). The statistical significance of differences between samples treated with Ca^2+^ and other treatment groups was also determined by one-way ANOVA **, *p* < 0.001 (*B*).

To further investigate the interaction between calmodulin and pro-IL-1β, a protein-binding assay was developed using ELISA-based methodology. In these experiments, immunoplates were coated with various concentrations of calmodulin or BSA control proteins, and the plates were probed with pro-IL-1β or mature IL-1β. The presence of bound IL-1β was detected using an HRP-labeled antibody and visualized using a chromogenic substrate. Consistent with the microarray data, there was a significant dose-dependent interaction between calmodulin and pro-IL-1β (Fig. 2*A*). Importantly, no interaction between pro-IL-1β and the control protein BSA was detected, suggesting that the binding is specific and robust. Interestingly, there was also no interaction between calmodulin and mature IL-1β, indicating that calmodulin is selective for the pro-form of the protein.

Because interactions between calmodulin and target proteins are classically facilitated by a calcium ion-driven conformational change (28), it was of interest to determine the calcium dependence of the interaction between pro-IL-1β and calmodulin. In these experiments, the interaction between pro-IL-1β and calmodulin was ablated completely when the binding assay was performed in calcium-free medium (Fig. 2*B*). To support these data, addition of the metal ion-chelating agents EDTA or EGTA for the total duration of the incubation with pro-IL-1β also ablated the interaction (Fig. 2*B*). Interestingly, addition of EDTA or EGTA for the final 10 min of incubation partially abrogated the interaction, suggesting that the interaction between pro-IL-1β and calmodulin is calcium-sensitive even after the pro-IL-1β has bound.

To confirm the apparent selectivity of calmodulin for pro-IL-1β and not the mature form, it was important to demonstrate that the detection antibody used in the protein-binding assay could detect both pro-IL-1β and mature IL-1β with equal avidity. It was also important to demonstrate that the removal of calcium from the reagent diluent was without nonspecific effects on the detection of pro-IL-1β. To assess this, plates were coated with an anti-IL-1β antibody overnight. The plates were then incubated with serial doubling dilutions (0.58–300 pm) of recombinant mature IL-1β, recombinant pro-IL-1β, recombinant pro-IL-1β in reagent diluent without calcium, recombinant pro-IL-1β in reagent diluent with EDTA, or recombinant pro-IL-1β in reagent diluent with EGTA. The amount of cytokine bound was detected using the standard secondary anti-IL-1 β reagents. In these experiments, neither the addition of the chelators (data not shown) nor the removal of calcium had any effect on the avidity of the binding assay for pro-IL-1β (Fig. 2*C*). However, these data did demonstrate that the anti-IL-1β reagents have greater avidity for the mature cytokine, relative to the 31-kD precursor, on a mole-per-mole basis. Given that it is pro-IL-1β and not mature IL-1β that has been shown to interact with calmodulin, the observed interaction with the precursor form cannot be reconciled on the basis of differential binding of the cytokine detection reagents. Together, these data provide strong evidence to suggest that pro-IL-1β, and not mature IL-1β, interacts with calmodulin in the presence of high calcium ion concentrations.

##### IL-1β Processing Is Attenuated by Calmodulin Inhibition

**FIGURE 3.**
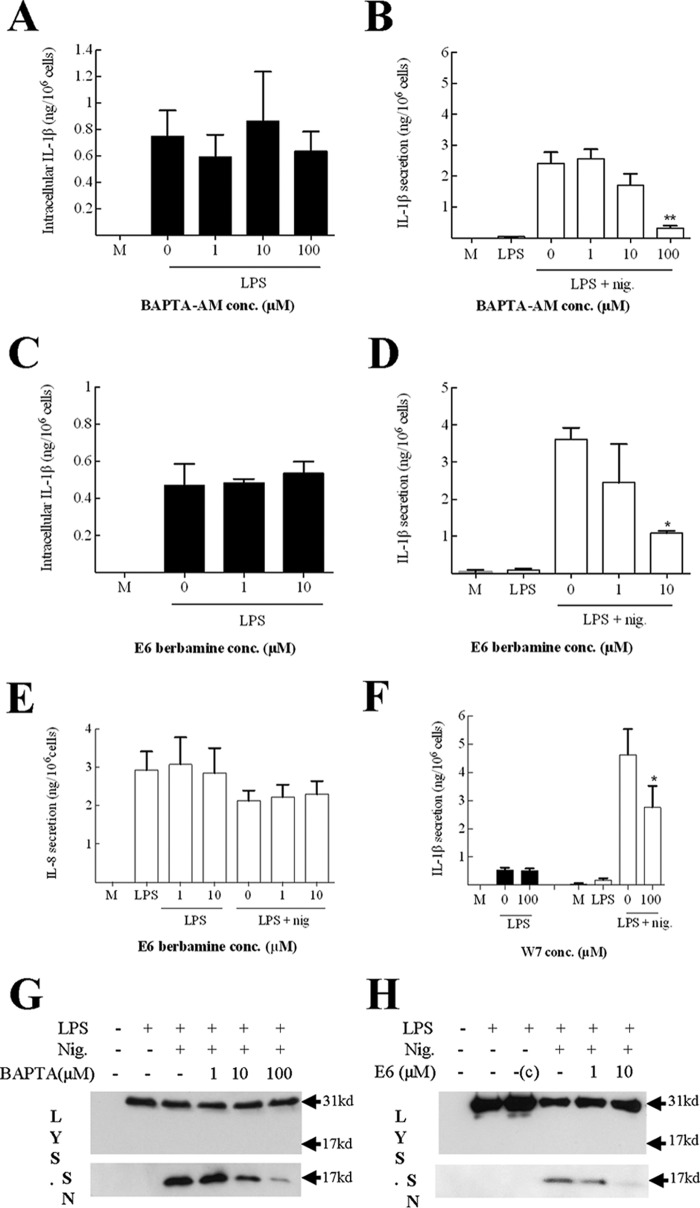
**Calcium chelation and calmodulin inhibition attenuate the cleavage of pro-IL-1β in THP-1 cells.** 10^6^ THP-1 cells (10^6^ cells/ml) were incubated with medium or primed with LPS (1 μg/ml) for 4 h, with the final 15 min in the presence or absence of BAPTA-AM (1, 10, or 100 μm; *A*, *B*, and *G*) or W7 (100 μm, *F*) or the final 30 min in the presence or absence of E6 berbamine (1 or 10 μm; *C–E* and *H*) or dimethyl sulfoxide control (*c*). These cells were then incubated for another hour in the presence or absence of nigericin (*nig*, 10 μm). Supernatants and lysates were then harvested and analyzed for the presence of IL-1β or IL-8 using cytokine-specific ELISAs. The IL-1β content of cell lysates (intracellular IL-1β) is displayed in *A*, *C*, and *E*, and the IL-1β content of supernatants (secreted IL-1β) is displayed in *B*, *D*, and *F*. The IL-8 content of selected supernatants is shown in *E*. Data shown are mean ± S.E. (*n* = 3). The statistical significance of differences between samples treated with LPS and nigericin and other treatment groups (*B*, *D*, and *F*) was determined by one-way ANOVA (*, *p* < 0.05; **, *p* < 0.001). Selected supernatants (*SN*) and lysates (*LYS*) were also analyzed by Western blot analysis using an anti-IL-1β antibody. A protein marker lane (*M*) on each gel was used to determine molecular weight. The blots were cropped in each case. *conc*, concentration.

To explore the function of the pro-IL-1β and calmodulin interaction, the role of calcium and calmodulin in IL-1β processing and secretion was investigated *in vitro*. Initial experiments confirmed that the human THP-1 monocytic cell line conformed to the current paradigm of IL-1 secretion, requiring two signals. Here, a 4-h incubation with the TLR4 ligand LPS induced a potent up-regulation of intracellular pro-IL-1β (Fig. 3*A*). Furthermore, stimulation of LPS-primed THP-1 cells with the ionophore nigericin for 1 h induced the processing and release of the cytokine. Using this experimental system and the intracellular calcium chelator BAPTA-AM, we first explored the role of calcium in IL-1β up-regulation and release. In these experiments, the addition of BAPTA-AM had no effect on the intracellular up-regulation of the pro-protein (Fig. 3*A*). However, the addition of BAPTA-AM (100 μm) almost completely ablated nigericin-stimulated IL-1β secretion (Fig. 3*B*). Importantly, the addition of BAPTA-AM had no significant effect on the viability of the THP-1 cells (data not shown). These data support the previous evidence (24), which suggests that IL-1β secretion requires the release of intracellular calcium stores.

To investigate the role of calmodulin in IL-1β production and secretion, the calmodulin inhibitor E6 berbamine was used. In these investigations, calmodulin inhibition had no effect on intracellular IL-1β protein expression (Fig. 3*C*). In contrast, the addition of E6 berbamine resulted in a significant (*p* < 0.05), dose-dependent inhibition in nigericin-induced IL-1β secretion (Fig. 3*D*). LPS-induced IL-8 secretion was unaffected by the presence of E6 berbamine across the same concentration range, suggesting that calmodulin is required specifically for IL-1β secretion and not for the secretion of cytokines in general (Fig. 3*E*). As with BAPTA-AM, E6 berbamine was without significant effect on the viability of THP-1 cells under the conditions used (data not shown). A similar pattern was observed with an alternative calmodulin inhibitor, W7, which also significantly reduced nigericin-induced secretion (Fig. 3*F*). Analysis of the BAPTA-AM- and E6 berbamine-treated samples by Western blotting revealed that both drugs dose-dependently inhibited the accumulation of mature (17-kD) IL-1β in the supernatants (Fig. 3, *G* and *H*). Because there was no evidence of mature IL-1β accumulation in the lysate, these data indicate that calmodulin inhibition attenuates IL-1β cleavage and not the secretion process.

**FIGURE 4.**
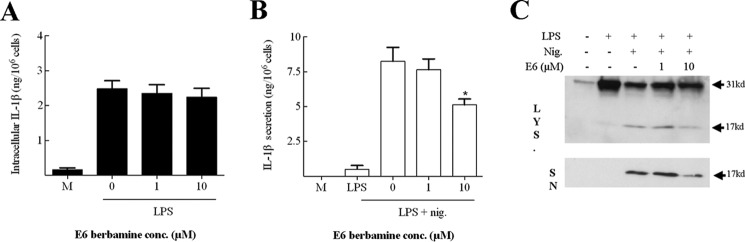
**Calmodulin is also required for secretion of IL-1β in human monocytes.** 10^6^ primary human monocytes (10^6^ cells/ml) were incubated with medium or primed with LPS (1 ng/ml) for 4 h, with the final 30 min in the presence or absence of E6 berbamine (1 or 10 μm). These cells were then incubated for another hour in the presence or absence of nigericin (*nig*, 10 μm). Supernatants and lysates were then harvested and analyzed for the presence of IL-1β using a cytokine-specific ELISA. The IL-1β content of cell lysates (intracellular IL-1β) is displayed in *A*, and supernatants (secreted IL-1β) are displayed in *B*. The data shown are mean ± S.E. (*n* = 4). The statistical significance of differences between samples treated with LPS and nigericin and other treatment groups was determined by one-way ANOVA (*, *p* < 0.05). Supernatants (*SN*) and lysates (*LYS*) were also analyzed by Western blotting using an anti-IL-1β antibody. *C,* a protein marker lane (*M*) on each gel was used to determine molecular weight. The blots were cropped in each case. *conc*, concentration.

To confirm that the effect of calmodulin inhibition on IL-1β processing was a feature of cells other than the THP-1 cell line, human primary peripheral blood monocytes were utilized. As with the THP-1 cells, LPS was used to up-regulate IL-1β expression, nigericin was used to stimulate cleavage and secretion of the pro-protein, and E6 berbamine was utilized to block calmodulin. Importantly, calmodulin inhibition had no effect on LPS induced up-regulation of intracellular IL-1β (Fig. 4*A*). In addition, and in concordance with the THP-1 data, calmodulin inhibition significantly down-regulated nigericin-stimulated IL-1β secretion (Fig. 4*B*). Finally, analysis of these samples by Western blotting demonstrated that E6 berbamine inhibited the cleavage of pro-IL-1β in human primary peripheral blood monocytes (Fig. 4*C*). Together, these data show that calmodulin inhibition attenuates nigericin-induced IL-1β secretion in monocytes, suggesting that calmodulin is an important protein in the processing and release of IL-1β.

##### Calmodulin Inhibition Has No Effect on the Assembly of the Inflammasome or the Activation of Caspase-1

**FIGURE 5.**
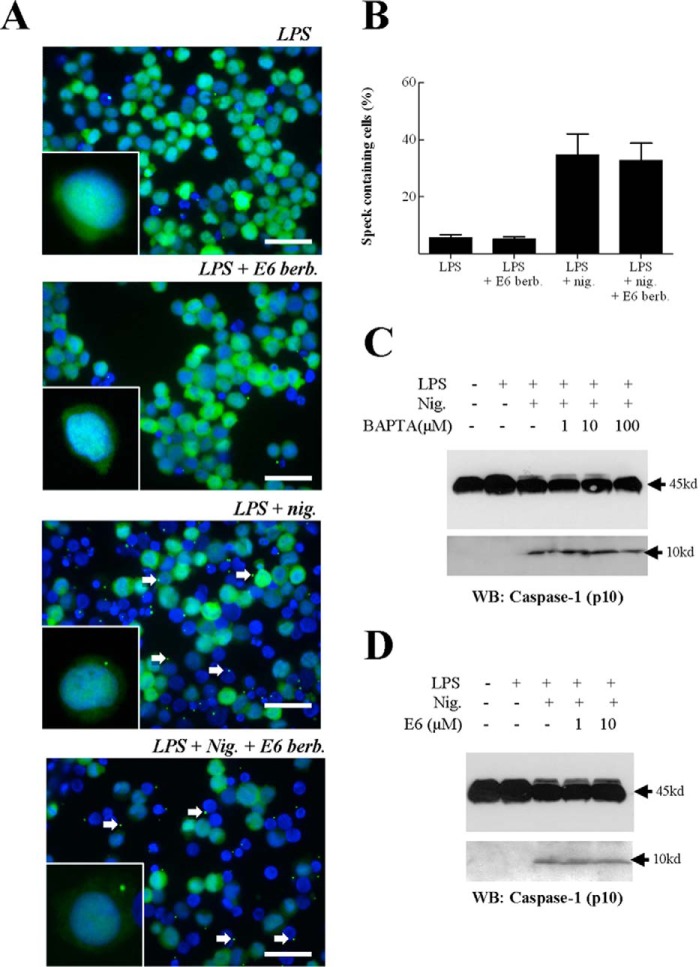
**Calmodulin inhibition has no effect on the assembly of the inflammasome or caspase-1 activation.** THP-1 cells (10^6^ cells/ml) were incubated with LPS (1 μg/ml) for 4 h, with the final 30 min in the presence or absence of E6 berbamine (*E6 berb*, 10 μm, *A* and *B*). These cells were then incubated for another hour in the presence or absence of nigericin (*nig*, 10 μm). Cells were then fixed and analyzed for ASC expression by immunofluorescence staining, with representative images taken. The number of speck-containing cells was quantified as a percentage of the total number of cells (*n* = 3, *B*). In addition, 10^7^ THP-1 cells (10^6^ cells/ml) were incubated with medium or primed with LPS (1 μg/ml) for 4 h, with the final 15 min in the presence or absence of BAPTA-AM (1, 10, or 100 μm; *C*) or the final 30 min in the presence or absence of E6 berbamine (1 or 10 μm, *D*). Supernatants were then harvested and analyzed by Western blotting (*WB*) using an anti-caspase-1 antibody. A protein marker lane on each gel was used to determine molecular weight. The blots were cropped in each case. *Scale bar* = 50 μm.

Having shown that calmodulin inhibition leads to an inhibition in IL-1β secretion, the role of calmodulin in this process was investigated further. Given that our data show that pro-IL-1β binds with calmodulin in a calcium-dependent interaction and that that calcium is required for processing, it is likely that the interaction between calmodulin and IL-1β is itself important for IL-1β processing and release. However, before this conclusion could be drawn, it was important to exclude the possibility that calmodulin inhibition was attenuating the assembly of the inflammasome because this would also explain why calmodulin inhibition leads to an inhibition in IL-1β secretion. To investigate the effect of calmodulin inhibition on inflammasome formation, nigericin-induced ASC speck formation in THP-1 cells was assessed (Fig. 5*A*). As reported previously, ASC expression was diffuse and throughout the cytoplasm in resting cells, whereas a single localized spot of ASC staining per cell was observed in cells in which inflammasome assembly had been provoked (29). Here the percentage of speck-containing cells was determined and used as a measure of inflammasome assembly (Fig. 5*B*). In the LPS-primed treatment group, there were very few cells with specks (∼5%), and this proportion was unaffected by the addition of the calmodulin inhibitor. As expected, the addition of nigericin to LPS-primed cells caused a large increase in the number of speck-positive cells (∼35%). Importantly, the addition of the calmodulin inhibitor had no effect on nigericin-induced speck formation, suggesting that calmodulin inhibition does not inhibit the assembly of the inflammasome.

To support this, the effect of inhibiting intracellular calcium release or calmodulin on caspase-1 activation was also investigated using an anti-caspase (p10) Western blot. As expected, all cells expressed high levels of the 45-kD proenzyme (Fig. 5, *C* and *D*). Addition of nigericin to LPS-primed THP-1 cells caused the accumulation of processed (10-kD) caspase-1 in both experiments. Importantly, incubation of cells with BAPTA-AM or E6 berbamine had no effect on nigericin-induced accumulation of processed caspase-1. Together, these data indicate that neither calmodulin nor the release of intracellular calcium are required for the activation of caspase-1.

##### Calmodulin Translocates within the Cytoplasm in Response to Nigericin

**FIGURE 6.**
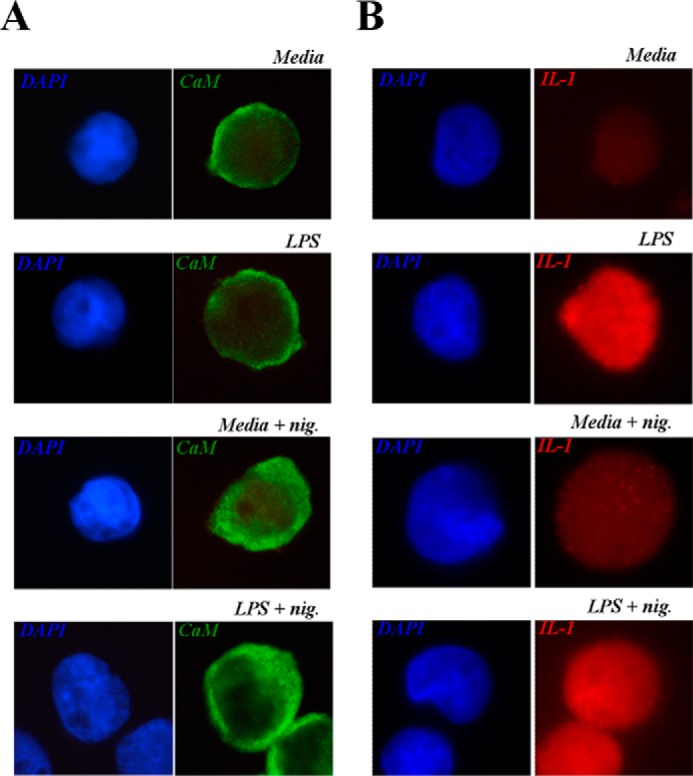
**Calmodulin, but not IL-1β, translocates within the cytoplasm in response to nigericin.** THP-1 cells (10^6^ cells/ml) were also incubated with media or LPS (1 μg/ml) for 4 h, with the final 30 min in the presence or absence of nigericin (*nig*, 10 μm). Cells were fixed and analyzed for the expression of IL-1β (*A*) or calmodulin (*B*) by immunofluorescence staining, and representative images were taken.

Despite considerable academic effort, the mechanisms and cellular locations of IL-1β maturation and release are still unclear. In light of the evidence presented previously, we speculate that calmodulin may play a vital role in orchestrating the events leading up to the processing of IL-1β, as induced by NLRP3 activators such as nigericin. To investigate this further, this study investigated how LPS priming and nigericin stimulation affected the intracellular localization of calmodulin in THP-1 cells (Fig. 6*A*). In untreated cells and in LPS-primed cells, the localization of calmodulin appeared to be focused in and around the intracellular side of the cell membrane. Interestingly, addition of 10 μm nigericin to both untreated and LPS-primed cells caused a rapid translocation of calmodulin throughout the cytoplasm. Together, these data suggest that nigericin has a profound effect on the intracellular localization of calmodulin. For completeness, we also investigated the cellular location of IL-1β under the same conditions. As expected, intracellular IL-1β levels were found to be elevated strongly in the cytosol of primed THP-1 cells relative to unprimed cells (Fig. 6*B*). However, unlike calmodulin, IL-1β appears to be already dispersed throughout the cytoplasm following expression. Together, these observations indicate that calmodulin may translocate toward cytosolic IL-1β in response to nigericin stimulation. However, further work is required to decipher the precise role of calmodulin in IL-1β processing.

## Discussion

In general terms, the processing and release of IL-1β is fundamental to the initiation and orchestration of inflammation. IL-1β is widely considered to be an essential effector in host defense responses, and its dysregulation is implicated in a multitude of pathologies (30–33). The mechanisms behind IL-1β secretion may represent significant therapeutic targets and are therefore of considerable academic interest. However, despite this interest, these processes remain poorly understood. In this study, we utilized a human proteome microarray containing 19,951 unique proteins to identify, globally, proteins that bind human recombinant pro-IL-1β. The rationale here was that the identification of such proteins may help elucidate the mechanisms of IL-1β release.

From the microarray, a number of potentially important pro-IL-1β-interacting proteins were identified, including POGZ and IL-22Rα2. IL-22Rα2 is a secreted inhibitor of IL-22, a cytokine involved in the initiation of innate immune responses (34, 35). Therefore, it is tempting to speculate that pro-IL-1β may serve to enhance IL-22-induced innate immune responses by inhibiting IL-22Rα2. However, because IL-22Rα2 is a secreted protein, it may be that the mature IL-1β plays more of a functional role here. POGZ is a poorly characterized intracellular protein that has been shown previously to interact with specificity protein 1 (SP1), a transcription factor involved in modulating gene expression (36). Because SP1 has been shown to act at the same sites as NF-κB, the interaction between POGZ and pro-IL-1β could have a role to play in modulating IL-1β gene expression.

Of the six hits identified on the microarray, both calmodulin and PLCXD3 are associated with intracellular calcium signaling. As discussed previously, the importance of calcium in IL-1β release is well established. In Brough *et al.* (24), ATP- and-nigericin induced IL-1β secretion was associated with a marked elevation in intracellular calcium ion concentration in murine macrophages. Importantly, the release of calcium from intracellular stores was shown to be required for IL-1β release in that study. In more recent investigations, inhibition of the pathways that lead to the release of intracellular Ca^2+^ stores, either by blocking inositol 1,4,5-trisphosphate-gated Ca^2+^ release channels, store-operated Ca^2+^ entry, or phospholipase C, effectively inhibited IL-1β processing (37). In this study, the addition of the intracellular calcium chelator BAPTA-AM inhibited nigericin-induced IL-1β processing and release in human monocytes. Together, these studies demonstrate a significant role for calcium in the secretion of IL-1β.

Having established that calcium has a vital role to play in IL-1β release, the imperative now is to decipher the mechanisms involved. It has been suggested in previous investigations that calcium is important for the activation of caspase-1 (37, 38). However, in this study, it was shown that the inhibition of intracellular calcium release did not have a marked effect on the nigericin-induced activation of caspase-1. Because the initial data presented here highlight that pro-IL-1β binds to calmodulin in a calcium-dependent interaction, it was hypothesized that damage-associated molecular pattern-induced increases in intracellular Ca^2+^ cause a conformational change in calmodulin, facilitating both the interaction between calmodulin and pro-IL-1β and, consequently, the processing of pro-IL-1β. In this investigation, it was demonstrated that calmodulin is important for nigericin-induced IL-1β cleavage and secretion in both the THP-1 cell line and in primary human monocytes. Crucially, the inhibition of calmodulin did not have a significant effect on nigericin-induced inflammasome assembly or caspase-1 activation. Together, these data support the hypothesis that the release of intracellular calcium is required for IL-1β processing because it facilitates an interaction between pro-IL-1β and calmodulin.

Although it was demonstrated here that the secretion of IL-8 is unaffected by calmodulin inhibition and that, therefore, calmodulin was not required for cytokine release *per se*, it cannot be discounted that the processing of other IL-1 cytokines is similarly dependent on calmodulin. This is a possibility given that many of these other IL-1 family members share high sequence homology, mechanisms of processing, and pathways of secretion. Therefore, it is suggested here that the role of calmodulin in the processing of other IL-1 cytokines should be subject to further investigation.

To explore the potential roles of the calmodulin/pro-IL-1β interaction in more detail, it is necessary to consider current hypotheses regarding pro-IL-1β processing together with the established roles of calmodulin. Intriguingly, calmodulin has been shown to bind to numerous intracellular proteins associated with a variety of processes, including inflammation, apoptosis, muscle contraction, intracellular movement, memory, nerve growth, and the immune response (39). Therefore, it is apparent that the role of calmodulin is dependent on the protein with which it interacts. It is well established that calmodulin can function as a coenzyme for a variety of inactive enzymes, including kinases and phosphatases (40, 41). In these cases, the interaction between calmodulin and its target results in the formation of an active enzyme or holoenzyme. As an example, calmodulin binds to inactive myosin light chain kinase upon increasing calcium concentrations and forms an active complex capable of phosphorylating myosin light chain during muscle contraction (42, 43). However, given that no evidence exists to suggest that pro-IL-1β exhibits enzymatic activity, it is unlikely that the observed calmodulin/pro-IL-1β interaction functions in this capacity.

More recently, evidence has suggested that calmodulin plays a role in the secretion of small secretory proteins. Specifically, Shao and Hegde (44) have demonstrated that calmodulin acts as an important chaperone in the translocation of these small proteins through the classic secretory pathway. It has also been shown that the interaction between calmodulin and small secretory proteins resulted in protection from protein aggregation and degradation. It is therefore tempting to speculate that calmodulin has a role to play in trafficking pro-IL-1β to caspase-1 for cleavage, especially given that the data presented here indicate that the calmodulin and pro-IL-1β interaction is required for IL-1β processing. Specifically, we suggest that calmodulin may play a role either in transporting the precursor toward caspases or presenting the precursor to caspases for proteolytic cleavage. Although these hypotheses are strengthened by the fact that calmodulin appears to translocate within the cytoplasm in response to nigericin, further investigation is required to determine the role of calmodulin in IL-1β release.

In addition to clarifying the role of the calmodulin-IL-1β interaction, the efforts to investigate the pathways and mechanisms involved in IL-1β processing are ongoing (45). It is becoming increasingly apparent that these pathways and mechanisms are remarkably complex, implicating a vast array of proteins, including messenger proteins, chaperones, proteases, ubiquitin ligases, cytosolic sensor molecules, and membrane-bound proteins (46). This study highlights that, by utilizing a more global, proteomic approach to determine the interactome of pro-IL-1β, we were able to elucidate a novel interaction that appears to be implicated in IL-1β secretion. This suggests that the use of further proteomic analyses may be of great benefit in determining the intricate processes that leads to the maturation and release of IL-1β. Specifically, analyzing the interactome of mature IL-1β or other IL-1 cytokines, such as IL-1α, IL-18, or IL-33, could reveal numerous, potentially important novel interactions and mechanisms in this field.

To conclude, this investigation highlights a number of pro-IL-1β-interacting proteins that may be relevant in elucidating the mechanisms of pro-IL-1β processing and secretion. We demonstrate that calmodulin, a ubiquitously expressed, calcium-sensitive protein, binds to pro-IL-1β in an interaction that is dependent on calcium. Moreover, we show that calmodulin is also important in the processing and release of pro-IL-1β, suggesting that this interaction is a key facet in the secretion of IL-1β.

## Author Contributions

J. S. A. conceived the study, coordinated the study, and wrote the paper. G. F. G., I. K., and R. J. D. provided assistance with the experimental design and interpretation and contributed to the preparation of the figures. All authors reviewed and approved the final version of the manuscript.

## Footnotes

2 The abbreviations used are:

ASC: apoptosis speck-like protein containing a CARD
NLR: NOD-like receptor
SNR: signal-to-noise ratio
ANOVA: analysis of variance.
